# The 5′ Untranslated Region of the *EFG1* Transcript Promotes Its Translation To Regulate Hyphal Morphogenesis in *Candida albicans*

**DOI:** 10.1128/mSphere.00280-18

**Published:** 2018-07-05

**Authors:** Prashant R. Desai, Klaus Lengeler, Mario Kapitan, Silas Matthias Janßen, Paula Alepuz, Ilse D. Jacobsen, Joachim F. Ernst

**Affiliations:** a Department Biologie, Molekulare Mykologie, Heinrich-Heine-Universität, Düsseldorf, Germany; b Microbial Immunology, Leibniz Institute for Natural Product Research and Infection Biology (Hans Knöll Institute), Jena, Germany; c Institute for Microbiology, Friedrich-Schiller-University, Jena, Germany; d Departamento de Bioquímica y Biología Molecular, Universitat de València, Burjassot, Valencia, Spain; e ERI Biotecmed, Universitat de València, Burjassot, Valencia, Spain; Carnegie Mellon University

**Keywords:** 5′ UTR, Candida albicans, *EFG1*, filamentation, hyphal morphogenesis, posttranscriptional regulation, translation

## Abstract

Extensive 5′ untranslated regions (UTR) are a hallmark of transcripts determining hyphal morphogenesis in Candida albicans. The major transcripts of the *EFG1* gene, which are responsible for cellular morphogenesis and metabolism, contain a 5′ UTR of up to 1,170 nucleotides (nt). Deletion analyses of the 5′ UTR revealed a 218-nt sequence that is required for production of the Efg1 protein and its functions in filamentation, without lowering the level and integrity of the *EFG1* transcript. Polysomal analyses revealed that the 218-nt 5′ UTR sequence is required for efficient translation of the Efg1 protein. Replacement of the *EFG1* open reading frame (ORF) by the heterologous reporter gene *CaCBGluc* confirmed the positive regulatory importance of the identified 5′ UTR sequence. In contrast to other reported transcripts containing extensive 5′ UTR sequences, these results indicate the positive translational function of the 5′ UTR sequence in the *EFG1* transcript, which is observed in the context of the native *EFG1* promoter. It is proposed that the 5′ UTR recruits regulatory factors, possibly during emergence of the native transcript, which aid in translation of the *EFG1* transcript.

**IMPORTANCE** Many of the virulence traits that make Candida albicans an important human fungal pathogen are regulated on a transcriptional level. Here, we report an important regulatory contribution of translation, which is exerted by the extensive 5′ untranslated regulatory sequence (5′ UTR) of the transcript for the protein Efg1, which determines growth, metabolism, and filamentation in the fungus. The presence of the 5′ UTR is required for efficient translation of Efg1, to promote filamentation. Because transcripts for many relevant regulators contain extensive 5′ UTR sequences, it appears that the virulence of C. albicans depends on the combination of transcriptional and translational regulatory mechanisms.

## Abstract (precis)

Many of the virulence traits that make Candida albicans an important human fungal pathogen are regulated on a transcriptional level. Here, we report an important regulatory contribution of translation, which is exerted by the extensive 5′ untranslated regulatory sequence (5′ UTR) of the transcript for the protein Efg1, which determines growth, metabolism, and filamentation in the fungus. The presence of the 5′ UTR is required for efficient translation of Efg1, to promote filamentation. Because transcripts for many relevant regulators contain extensive 5′ UTR sequences, it appears that the virulence of C. albicans depends on the combination of transcriptional and translational regulatory mechanisms.

## INTRODUCTION

Transcriptional networks are known to govern growth and virulence of the human fungal pathogen Candida albicans. Transcription factors have been identified that regulate the interconversion between its yeast cell form and a filamentous hyphal form, or the rod-like opaque form. Efg1 is a key basic-helix-loop-helix (bHLH)-type regulatory protein that controls hyphal morphogenesis in a dual manner, promoting filamentation under normoxia in the presence of environmental cues (1, 2) but repressing it under hypoxia (3, 4). Its promoting function depends on increased histone acetylation and chromatin remodeling at promoters of target genes (5), which facilitate hyphal initiation; shortly thereafter, however, *EFG1* expression is strongly downregulated to prevent its interference with subsequent processes required for hyphal formation (6, 7). Under hypoxia, Efg1 represses the expression of genes encoding hyphal inducers Ace2 and Brg1, thereby downregulating filamentation (8), and it regulates the hypoxia-specific expression of numerous genes. Furthermore, by counteracting expression of *WOR1*, Efg1 prevents switching to the opaque form and favors the yeast morphology (9). The activity of the Efg1 protein is regulated by posttranslational modifications, including phosphorylation by cAMP-dependent protein kinase A (PKA) in response to environmental cues (10, 11). The overall activity of Efg1 is required for biofilm formation (12–14) and virulence (2) of C. albicans.

In eukaryotes, the level, processing, localization, and/or structure of the primary transcript determines the initial amount of the encoded protein, which is subsequently lowered by different rates of proteolytic degradation. Some such posttranscriptional processes and their underlying mechanisms have been described in C. albicans to regulate levels of proteins, including transcription factors (15, 16). Transcript degradation involves poly(A) tail removal by deadenylase subunits Ccr4/Pop2 (17), hydrolysis of the 5′ cap by decapping activators Dhh1/Edc3 (18) and decapping enzyme Dcp1 (18), and mRNA digestion by exonuclease Xrn1/Kem1 (19, 20). RNA binding proteins Puf3 (21) and Zfs1 (22) also appear to be involved in decay of transcripts. Mutants lacking these degradative activities show defects in filamentation and/or biofilm formation, although specific targets have not yet been defined. The specific degradation of the transcript encoding Nrg1, a strong repressor of filamentation, was described to depend on an antisense transcript that originates from the locus encoding the Brg1 hyphal activator (23). The localization of transcripts also regulates filamentation of C. albicans, as was shown for the She3 protein that binds several transcripts involved in filamentation and transports them to the bud site of yeast cells or to the tips of hyphae (24); the Sec2 protein operating at the hyphal tip appears to specifically localize its own transcript to this location (25). It is assumed that localized translation procures directed delivery of such proteins to their sites of action. In recent years, the localization, degradation, and/or translation of certain transcripts was found also to depend on promoter sequences, suggesting that already during transcription, regulatory factors for these functions may become loaded onto the emerging transcript (26–28).

The structure of the 5′ untranslated region (UTR) of transcripts controls translation in eukaryotes. Strong evidence supports the importance of AUG context sequence on translational initiation (29, 30). Upstream open reading frames (uORFs) within the 5′ UTR can control translation of the downstream main ORF (31, 32), as has been described for the C. albicans* GCN4* gene that regulates the amino acid starvation response, as well as filamentation and biofilm formation (33). Cap-independent translation that is initiated at internal ribosome entry sites (IRESs) has been described for gene transcripts responsible for invasive growth in the yeast Saccharomyces cerevisiae (34). In addition, 5′ UTR sequences may contain binding sites for binding proteins that facilitate localization (35) and potentially translation of transcripts. In C. albicans, the Dom34 protein, known for its general role in no-go decay of mRNAs, was also shown to bind the 5′ UTR of specific transcripts encoding Pmt-type mannosyl transferases and favor their translation (36). Similarly, the Ssd1 RNA binding protein may positively affect translation of specific sets of transcripts involved in cell wall integrity and polarized growth (37, 38). Remarkably, many transcripts encoding essential regulators of cell morphology contain extensive 5′ UTRs, including *UME6* (3,041 nucleotides [nt]), *CZF1* (2,071 nt), *WOR1* (2,978 nt), and *EFG1* (1,139 nt of long transcript) (39). The long 5′ UTRs of *UME6* and *WOR1* genes were recently shown to downregulate translation of their transcripts (40, 41), possibly by forming a tight three-dimensional structure that blocks scanning by ribosomal 40S subunits. In both cases, regulated release of translational blockage may be mediated by host environmental cues that alter the 5′ UTR structure (42), e.g., in the presence of specific RNA binding proteins. Nonnative, functional expression of *EFG1* has been achieved by placing the *EFG1* ORF (without the 5′ UTR sequence) downstream of the heterologous C. albicans* PCK1* and *ACT1* promoters (1, 3, 43, 44). Here, we report that the extensive 5′ UTR of the major *EFG1* transcript nevertheless has a significant positive role for the functional expression of the *EFG1* ORF. A specific sequence within the 5′ UTR is required to stimulate translation of the *EFG1* transcript, to permit efficient hyphal morphogenesis.

## RESULTS

### Deletions in the 5′ UTR of *EFG1.*

**FIG 1:**
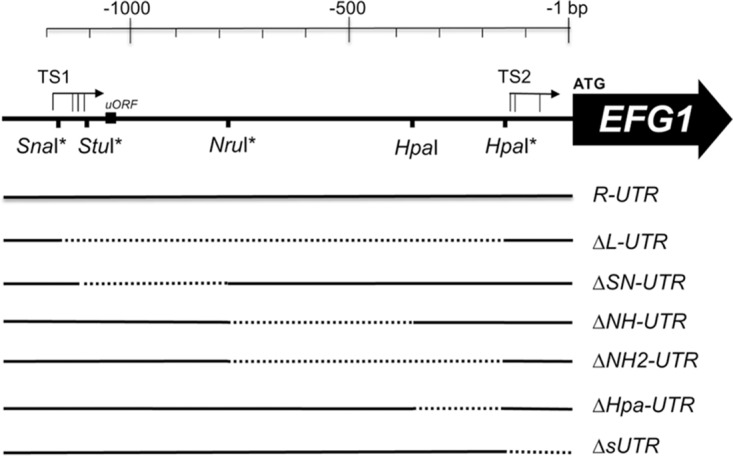
Upstream region of the *EFG1* gene. Schematic representation of the *EFG1* upstream region in strain ATCC 10231 indicating start positions of the large transcript (TS1) around position −1100 and of the small transcript (TS2) around position −100. The large and small transcripts are the major transcripts observed in white (yeast) and opaque growth forms, respectively (Fig. S1). A small upstream open reading frame (uORF) encoding 4 amino acids is shown as a black box; it is missing in strain SC5314. Positions of restriction sites used to construct deletions in the 5′ UTR sequence are as indicated; sites marked by asterisks were introduced by site-specific mutagenesis. While *R-UTR* denotes the full-length 5′ UTR-*EFG1* region, the Δ series shows deleted *EFG1* alleles lacking sequences between restriction sites in the 5′ UTR (dotted lines), affecting mostly the large transcript but also the small transcript (Δ*sUTR*). Plasmids harboring native and deleted forms of *EFG1* were integrated into the *EFG1* upstream region of *efg1*/*efg1* mutant HLC67.

In the yeast growth form (white), the transcript start sites for the main *EFG1* transcript are known to cluster around position −1100 relative to the ATG of the *EFG1* ORF, generating a transcript of 3.3 kb (6, 43, 45). Referring to the sequence of ATCC 10231 (used here for deletion analysis), start sites lie at positions −1170, −1143, and −1112 (amended from the work of Tebarth et al. [6]) or at −1125 (−1117 in strain SC5314 [39]); in agreement, the start site in strain WO-1 was mapped at position −1173 (45) (Fig. 1). In the rod-like opaque growth form, however, low levels of a shortened *EFG1* transcript of 2.2 kb occur (43), for which start sites at positions −145 and −162 were identified (45), and a start position of −74 was also observed for a minor fraction of the *EFG1* transcript in yeast-form cells (6).

10.1128/mSphere.00280-18.1 FIG S1  Upstream sequence of the *EFG1* ORF. The DNA sequence preceding the ATG start site of the *EFG1* gene in strain ATCC 10231 is shown. Sequences marked in red were altered to introduce novel cleavage sites for restriction enzymes (asterisks). These sites and the native HpaI site at position −386 to −391 were used for deletion construction. Transcription start sites are indicated by arrowheads: in the yeast growth form, start sites of the large transcript (3.3 kb) were identified at positions −1170, −1143, and −1112 and at position −1125, which corresponds to −1117 in strain SC5314. Start sites of a short transcript (2.2 kb) occurring in the yeast form lie at position −74 or, in the opaque growth form of strain WO-1, at positions −145 and −162. Sequences representing putative binding sites for the TATA box binding protein (TBP-box) or the Matα2 regulator protein are in green font. A short uORF encoding 4 amino acids, which occurs in strain ATCC 10231 but not in strain SC5314, is marked in blue font and is underlined. In addition, putative uORFs starting with a non-ATG sequence (TTG) are marked in blue and green italics, but only uORFs occurring in both ATCC 10231 and SC5314 strains are indicated. Download FIG S1, TIF file, 0.3 MB. Copyright © 2018 Desai et al. 2018 Desai et al. This content is distributed under the terms of the Creative Commons Attribution 4.0 International license.

To construct deletions in the 5′ UTR sequence, restriction enzyme sites were inserted, singly or in combination, into a plasmid-resident *EFG1* gene, including 3.2 kb of its upstream sequence (allele *R-UTR*). Sequences between restriction sites were deleted, resulting in six deleted *EFG1* alleles lacking 5′ UTR sequences of the large transcript (Δ*L*-, Δ*SN*-, Δ*NH*-, Δ*NH2*-, and Δ*Hpa-UTR*) or the small transcript (Δ*sUTR*) (Fig. 1; see also Fig. S1 in the supplemental material). The resulting plasmids were chromosomally integrated into the upstream region of the *EFG1* locus in strain HLC67 (2), which lacks the *EFG1* ORF (but retains its upstream sequences) on both homologous chromosomes.

### 5′ UTR sequence enhances filamentation.

**FIG 2:**
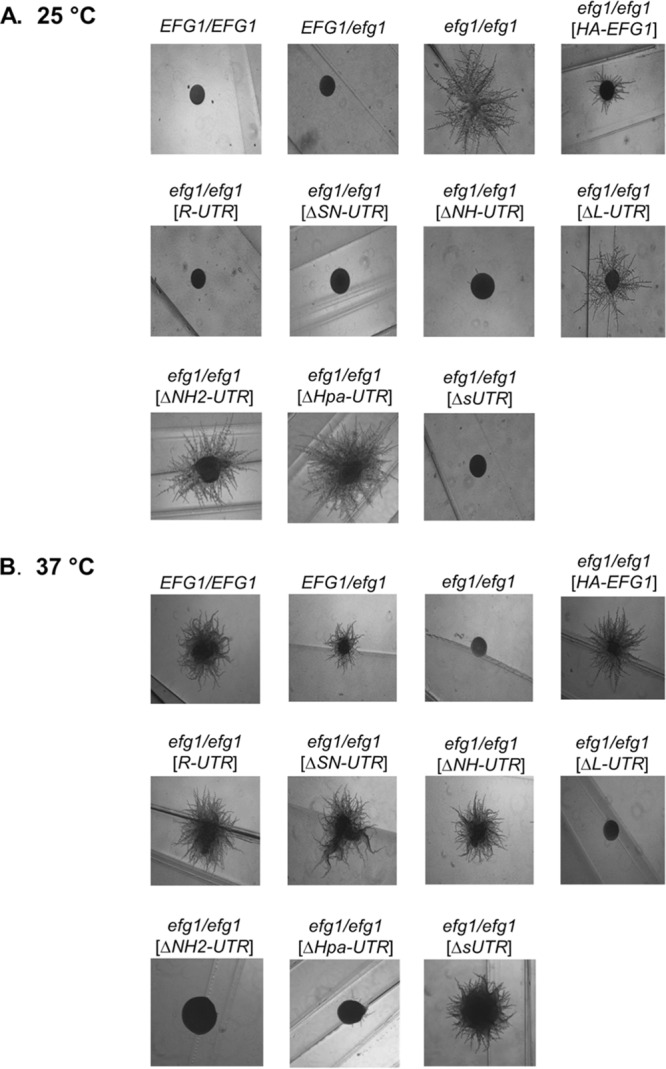
Colony phenotypes of strains expressing deletions in the 5′ UTR of *EFG1*. Strains CAF2-1 (*EFG1*/*EFG1*), BCA09 (*EFG1*/*efg1*), HLC67 (*efg1/efg1*), HLCEEFG1 (*HA*-*EFG1*/*efg1*), PDUWT (*efg1/R-UTR-EFG1*), PDUSN (*efg1/*∆*SN-UTR-EFG1*), PDUNH (*efg1/*∆*NH-UTR-EFG1*), PDULG (*efg1/*∆*L-UTR-EFG1*), PDUSH (*efg1/*∆*NH2-UTR-EFG1*), PDUHH (*efg1/*∆*Hpa-UTR-EFG1*), and PDUsU (*efg1/*∆*sUTR-EFG1*) were grown on Spider medium either at 25°C for 3 days (A) or at 37°C for 2 days (B), under hypoxic conditions (0.2% O_2_). The data show representative colony morphologies, which were imaged by light microscopy.

C. albicans mutants lacking the Efg1 protein are unable to form hyphae at 37°C under all conditions, while at temperatures of <35°C, if cells are grown under hypoxia on agar surfaces, their filamentation is derepressed (4). This dual function of Efg1 as activator and as repressor of morphogenesis becomes apparent during surface growth of cells under hypoxia (0.2% O_2_) at either 25°C or 37°C (Fig. 2). Cells carrying at least one functional *EFG1* allele are able to filament at 37°C but not at 25°C, while nonfunctional alleles are hyperfilamentous at 25°C but not at 37°C. The only exception to this pattern, as described previously (8), is mediated by the *HA-EFG1* allele, which promotes hypha formation at 37°C but lacks repressor function at 25°C, thus leading to filamentation at both temperatures.

**FIG 3:**
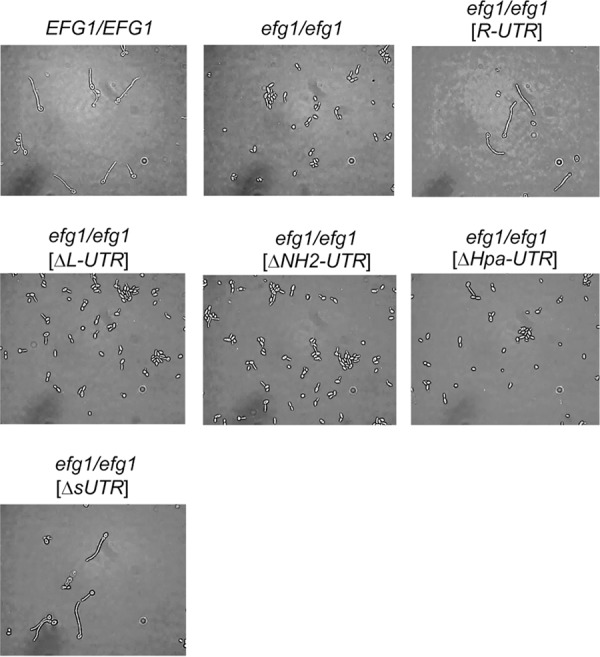
Cell morphologies of strains expressing deletions in the 5′ UTR of *EFG1* after serum induction. Strains were grown in YPD at 30°C and diluted into prewarmed YP medium containing 10% horse serum at 37°C. Cells were incubated for 30 min at 37°C and imaged by phase-contrast microscopy. Strain designations are as in Fig. 2.

**FIG 4:**
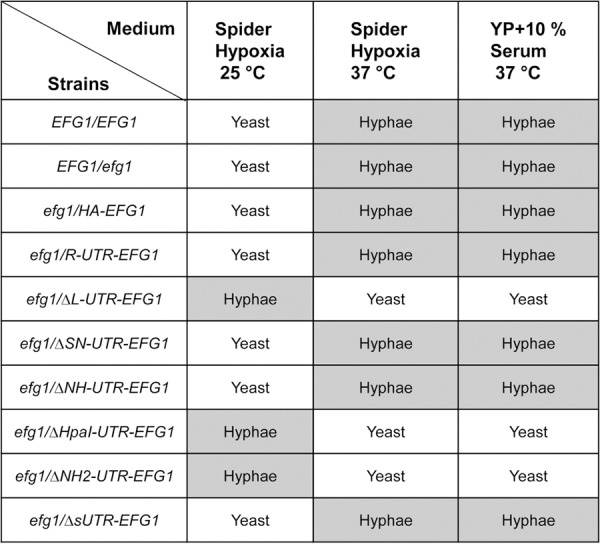
Summary of filamentation phenotypes of C. albicans strains carrying deletions in the 5′ UTR of *EFG1*.

*EFG1* alleles containing either the full-length 5′ UTR (*R-UTR*) or deleted alleles Δ*SN-UTR*, Δ*NH-UTR*, and Δ*sUTR* were fully active in promoting filamentation at 37°C and repressing it at 25°C (Fig. 2). Because deletions in these alleles encompassed a small uORF sequence, it appears that its presence is not required for hypha formation. In contrast, alleles containing Δ*L-UTR*, Δ*NH2-UTR*, and Δ*Hpa-UTR* performed as nonfunctional *EFG1* alleles that did not stimulate filamentation at 37°C but allowed strong filamentation at 25°C. The latter alleles were all lacking the 218-bp HpaI fragment that was solely deleted in the Δ*Hpa-UTR* allele. To confirm these results, the function of the various alleles was also tested under normoxia using liquid induction medium containing 10% serum at 37°C, which demonstrated similar filamentation phenotypes as those that were observed during surface growth (Fig. 3). Thus, these results indicate that the 218-nt HpaI fragment in the 5′ UTR of *EFG1* is required for production and/or activity of Efg1, promoting filamentation at 37°C and repressing it at 25°C. Filamentation phenotypes obtained for all tested *EFG1* alleles are summarized in Fig. 4.

### Deleted 5′ UTR alleles do not lower *EFG1* transcript levels.

**FIG 5:**
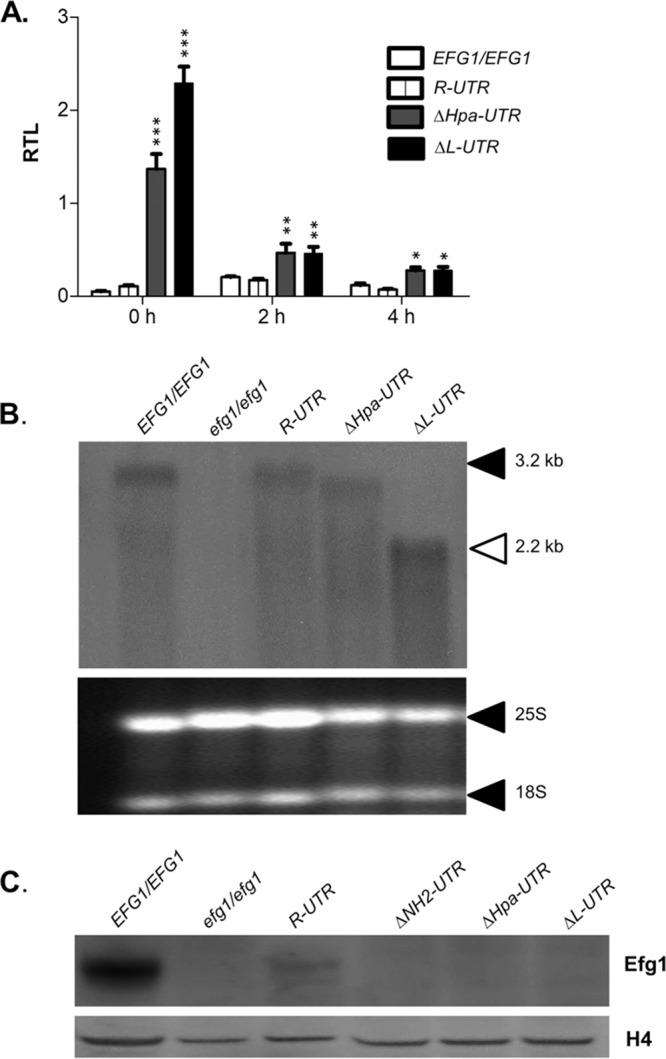
A 5′ UTR deletion increases *EFG1* transcript and decreases Efg1 protein levels. Strains CAF2-1 (*EFG1*/*EFG1*), PDUWT (*R-UTR*), PDUHH (∆*Hpa-UTR*), and PDULG (∆*L-UTR*) were examined for *EFG1* transcript and Efg1 protein levels. (A) Strains were pregrown for 12 h in YPD (*t* = 0 h), diluted into YPD, and grown for 2 and 4 h at 30°C; levels of the *EFG1* transcript were determined by qPCR, using the *ACT1* transcript as an internal reference to calculate relative transcription levels (RTL). Error bars display standard errors of the means derived from biological triplicates. A two-tailed, unpaired *t* test comparing the RTL values of control CAF2-1 and other strains was used to determine the statistical relevance. *, *P* < 0.05; **, *P* < 0.01; ***, *P* < 0.001. (B) In addition, the *EFG1* transcript in the RNA of strains grown for 6 h in YPD was examined by Northern analysis (top), using 8 µg RNA for loading. Note that the *EFG1* transcript size (3.2 kb) is reduced greatly only for the Δ*L-UTR* variant, which lacks most of the 5′ UTR. 25S (3.4-kb) and 18S (1.8-kb) rRNAs (61) stained by ethidium bromide were used as a loading control (bottom). (C) To determine Efg1 protein levels, strains were grown in YPD medium at 30°C to the logarithmic phase and cell extracts derived from 1 OD_600_ equivalent of cells were separated by SDS-PAGE and analyzed by immunoblotting, using either anti-Efg1 antibody or anti-histone H4 antibody for probing. Levels of histone H4 served as loading controls.

To clarify the reasons for the inactivity of *EFG1* alleles in cells lacking the 5′ UTR completely (Δ*L-UTR*) or partially (Δ*Hpa-UTR*), *EFG1* transcript levels were determined by quantitative PCR (qPCR). Both shortened alleles resulted in significantly elevated transcript levels compared to wild-type cells (*EFG1/EFG1*) or to cells expressing the *R-UTR* allele containing the full-length 5′ UTR (Fig. 5A). The observed increase was highest in cells pregrown for 12 h in yeast extract-peptone-dextrose (YPD) (*t* = 0) but clearly apparent also after short-term growth for 2 and 4 h. It can be concluded that the low Efg1 activity of the Δ*L-UTR* or Δ*Hpa-UTR* alleles cannot be explained by lowered* EFG1* transcript levels. To verify that the respective transcripts were intact, cellular RNA was also examined by Northern blotting. As expected, wild-type cells and cells containing the *R-UTR* allele contained an *EFG1* transcript of about 3.2 kb (6, 42, 44), while the *efg1* mutant was lacking this transcript (Fig. 5B). Remarkably, the mutated alleles encoded *EFG1* transcripts with sizes reflecting the extent of 5′ UTR deletions, i.e., the size of the transcript encoded by the Δ*Hpa-UTR* allele was only slightly reduced, while the Δ*L-UTR* transcript was shortened to about 2 kb, approximating the size that occurs in opaque-type cells (42, 44). These results indicate that the *EFG1* transcript encoded by the inactive, deleted 5′ *EFG1* alleles is not differentially processed or degraded.

### Efg1 protein produced by deleted 5′ UTR alleles.

To verify Efg1 protein levels produced by the deleted 5′ UTR alleles, cell extracts were analyzed by immunoblotting, using an anti-Efg1 antiserum described previously (7, 46). The Efg1 protein was detected strongly in wild-type cells (carrying two *EFG1* alleles) and also, with reduced intensity, in cells carrying a single *R-UTR* allele containing the full-length 5′ UTR (Fig. 5C). In contrast, no Efg1 protein was observed in cells expressing the truncated 5′ UTR versions Δ*L-UTR*, Δ*Hpa-UTR*, and Δ*NH2-UTR*, which are functionally inactive. It can be concluded that the latter alleles do not produce significant amounts of Efg1 protein, in spite of expressing high *EFG1* transcript levels.

### Truncation of the 5′ UTR deletion reduces translation of *EFG1.*

**FIG 6:**
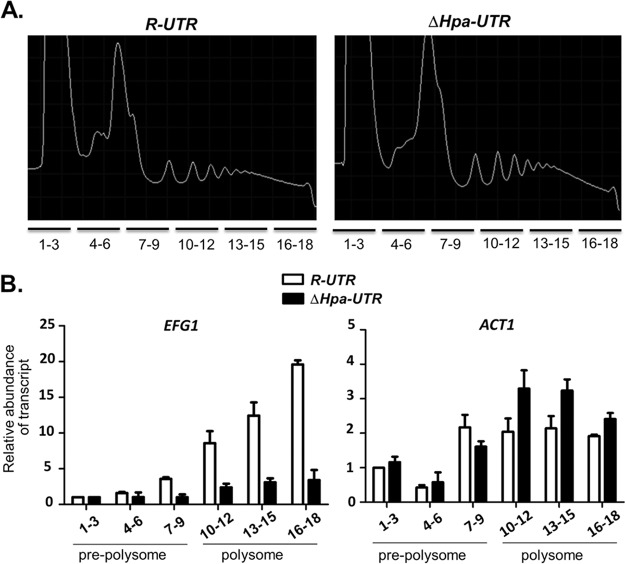
Transcript fractionation on polysome gradients. (A) Strains PDUWT (*R-UTR*) and PDUHH (*∆Hpa-UTR*), pregrown in YPD medium at 30°C, were transferred to YP medium containing 10% horse serum at 37°C and incubated for 15 min. Cellular extracts of strains were centrifuged in a 10 to 50% sucrose gradient, which was subsequently fractionated. Nucleic acids in gradient fractions were detected by absorbance (*A*_260_). Note that prepolysome fractions 1 to 9 contain 40S, 60S, and 80S rRNA. (B) *EFG1* and *ACT1* transcripts in gradient fractions were detected by qPCR after adding a known amount of an *in vitro*-generated transcript of the *CaCBGluc* gene as a calibrator. Data shown represent values that are normalized to *EFG1* or *ACT1* mRNA abundance in fraction 1. Each bar represents the normalized mean *EFG1* or *ACT1* transcript level of two independent experiments with three technical replicates and includes the standard error of the mean.

The above results had suggested that the 5′ UTR of the *EFG1* transcript contains a 218-nt sequence corresponding to the small HpaI fragment of the *EFG1* upstream region, which is required for efficient translation of Efg1. To test this hypothesis, polysome analyses were carried out using cellular lysates of strains expressing *EFG1* alleles containing the full-length 5′ UTR (*R-UTR*) or the partially deleted variant (Δ*Hpa-UTR*). As expected, profiles obtained by sucrose gradient centrifugation were similar in the two strains, showing a prepolysomal fraction (containing 40S, 60S, and 80S rRNA) and several polysomal peaks (Fig. 6A). Transcript levels of *EFG1* and the *ACT1* housekeeping gene in the prepolysomal and polysomal fractions were examined by qPCR, using a spiked-in control RNA as a reference. The results demonstrate that the *EFG1* transcript containing the full-length 5′ UTR is significantly enriched in the polysomal fraction compared to the prepolysomal fraction (Fig. 6B), while in cells expressing the Δ*Hpa-UTR* allele, the *EFG1* transcript occurred in similar amounts in pre- and polysomal fractions. In contrast, the *ACT1* transcript used as a control was increased in the polysomal fraction and occurred in similar amounts in the two types of cells (slightly increased in cells with the Δ*Hpa-UTR* allele). The results indicate that a specific deletion within the 5′ UTR of the *EFG1* transcript impairs its translation.

### ORF-independent function of the 5′ UTR sequence.

**FIG 7:**
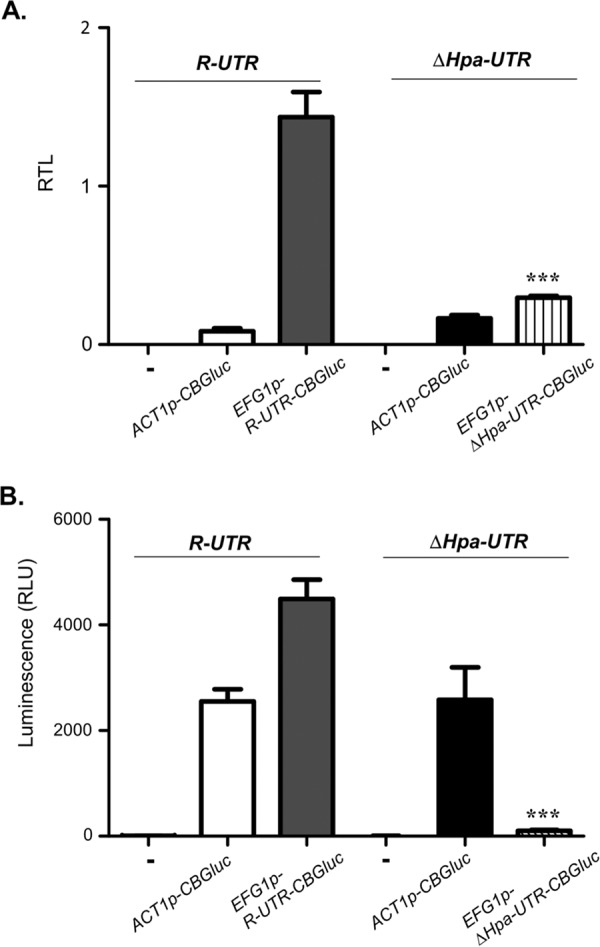
Deletions in 5′ UTR affect the translation of *CaCBGluc*. Strains containing either the intact *R-UTR* allele (strain PDUWT) or the Δ*Hpa-UTR* allele (strain PDUHH) of *EFG1* were modified further by replacing either the *ACT1* or *EFG1* ORF with the *CaCBGluc* ORF. Resulting strains include ACT1GN (*efg1/EFG1p-R-UTR-EFG1*, *ACT1*/*ACT1p*-*CaCBGluc*), EFG1N (*efg1/EFG1p-R-UTR-CaCBluc*), DUTRinACT1GN (*efg1*/*EFG1p-*∆*Hpa-UTR-EFG1*, *ACT1/ACT1p-CaCBGluc*), and DUTRinEFG1GN (*efg1*/*EFG1p-*∆*Hpa-UTR-CaCBGluc*). Strain PDUWT (*efg1/EFG1p-R-UTR-EFG1*) was used as a control (−). Strains were grown in YPD medium at 30°C to the exponential phase (OD_600_ of 0.5) and used for determining transcript and luciferase activity levels. (A) *CBGluc* transcript level. Relative transcript levels (RTL) of *CaCBGluc* were determined by qPCR, using the *ACT1* transcript for normalization. Error bars display standard errors of the means derived from three biological and three technical replicates. (B) Luciferase activity. Luminescence originating from 100 µl of cells was assayed after addition of 100 µl of Beetleglow reagent. Statistical significance was determined by comparing the EFG1GN and UTRinACT1GN strains, with a two-tailed, unpaired *t* test based on two biological and three technical replicates. *, *P* < 0.05; **, *P* < 0.01; ***, *P* < 0.001.

The observed positive effect of the 5′ UTR of the *EFG1* transcript on its translation could operate either independently or dependently on its native context upstream of the *EFG1* ORF. This possibility was examined by replacing the *EFG1* ORF in control strain PDUWT (*efg1/R-UTR-EFG1*) by the heterologous *CaCBGluc* sequence that encodes click beetle luciferase (47); thereafter, the resulting strain EFG1GN contained the allele *EFG1p*-*R-UTR-CaCBGluc*. Likewise, the *EFG1* ORF was replaced in strain PDUHH (*efg1*/Δ*Hpa-UTR-EFG1*), resulting in strain DUTRinEFG1GN containing allele *EFG1p*-Δ*Hpa-UTR-CaCBGluc*. As controls, the *CaCBGluc* gene was also used to replace one allele of the *ACT1* ORF in both PDUWT and PDUHH, generating strains ACT1GN and DUTRinACT1GN, which both carry the *ACT1p*-*CaCBGluc* fusion. *CaCBGluc* transcript levels driven by the *ACT1* promoter were similar in strains ACT1GN and DUTRinACT1GN, as expected (Fig. 7A); correspondingly, luciferase activities were nearly identical (Fig. 7B). Under the control of the *EFG1* promoter that was joined to the intact 5′ UTR (*R-UTR*), the *CaCBGluc* transcript level was about 5-fold higher than its junction to the deleted 5′ UTR sequence (allele Δ*Hpa-UTR*), suggesting that truncation of the 5′ UTR lowers transcript stability. It should be considered here that negative autoregulation known for the *EFG1* gene (Fig. 5) (6, 7) cannot occur for the described *CaCBGluc* fusions. Remarkably, however, in spite of considerable *CaCBGluc* transcript levels, luciferase activity was essentially lost in strain DUTRinEFG1GN. The complete loss of luciferase activity was surprising, considering that the *CaCBGluc* transcript level in this strain was even higher than in control strain DUTRinACT1GN (*CaCBGluc* transcribed by the *ACT1* promoter), which generated abundant luciferase activity. The results support the importance of the 5′ UTR *EFG1* sequence for the functional expression of the downstream ORF, which need not be the native *EFG1* ORF.

## DISCUSSION

The dual activity of Efg1 as an activator and repressor of transcription requires proper timing and targeting of its activity. Although Efg1 is required to initiate hypha formation under normoxia (1, 2), its prolonged activity interferes with orderly filamentation (6, 7). Under some hypoxic conditions, Efg1 is not an activator but an efficient repressor of hypha formation (3, 4). Efg1 induces genes specific for the yeast (white) growth form, but by repressing *WOR1*, it prevents the rod (opaque) growth form (9). In metabolism, Efg1 induces genes involved in glycolysis, but it also represses genes in oxidative metabolism (3). Furthermore, Efg1 induces and represses hypoxia-specific genes, and it prevents inappropriate hypoxic regulation of genes not normally regulated by oxygen (4). Efg1 activity has hitherto been known to be regulated on posttranslational and transcriptional levels. Posttranslational modes of regulation include Efg1 phosphorylation by PKA isoforms (10, 11), which may occur directly at target genes (48), or physical association with regulatory factors like Flo8 and Czf1 (46). Transcriptional repression of *EFG1* expression is mediated by Sin3 (6) and Wor1 (9) and also by Efg1 itself (6, 7), causing negative autoregulation that prevents an overshoot of Efg1 activity. *EFG1* activation is mediated in an environment-dependent manner by Brg1, Bcr1, or Ace2 (8). Here, we report a novel mechanism regulating Efg1 biosynthesis on the translational level.

We present evidence that a 218-nt sequence of the 5′ UTR of its major transcript is required for Efg1 protein production. Because of negative autoregulation of *EFG1* (6, 7), transcript levels of the 218-nt deletion variant were even increased but still did not yield significant amounts of Efg1 protein. In wild-type cells, the major *EFG1* transcript was distributed mostly to polysomes, while the deleted transcript was distributed equally to monosomes and polysomes, suggesting that the 218-nt sequence activates Efg1 translation. This positive effect was observed even if the *EFG1* ORF was replaced by the ORF of a heterologous reporter gene, indicating that the activating function of the 5′ ORF does not depend on its native 3′ context. As expected from these results, the absence of this regulatory sequence in the short 2.2-kb transcript of the opaque form (or in the minor 2.2-kb transcript of the white yeast form) (6, 43, 45) is expected to reduce the production of Efg1 protein. This mechanism contributes to lowering Efg1 activity in opaque cells, which is already reduced on the transcriptional level (9, 43, 45), to prevent backward switching to the white (yeast) form. Clearly, low translation of the *EFG1* transcript in opaque cells (41) is not caused by an inhibitory effect of the 5′ UTR, as has been suggested elsewhere (41), but is due to the lack of the 218-nt sequence in the short opaque transcript (2.2 kb). The positive translational function of the 5′ UTR in the *EFG1* major transcript differs from other recently reported 5′ UTRs in transcripts of two different C. albicans genes. In contrast to *EFG1*, 5′ UTR sequences of both *UME6* and *WOR1* transcripts were found to negatively influence translation of the respective proteins (40, 41). Furthermore, both *UME6* and *WOR1* are positively autoregulated (40, 41, 49), while *EFG1* is negatively autoregulated. The different modes of autoregulation nevertheless lead to increased promoter activities and transcript levels of all three genes lacking the 5′ UTR (or relevant parts thereof); in the case of *UME6/WOR1*, this result is caused by relief of translational inhibition (increased protein levels stimulate promoter activity), while for *EFG1* this occurs because Efg1 production is reduced, which derepresses *EFG1* promoter activity.

**FIG 8:**
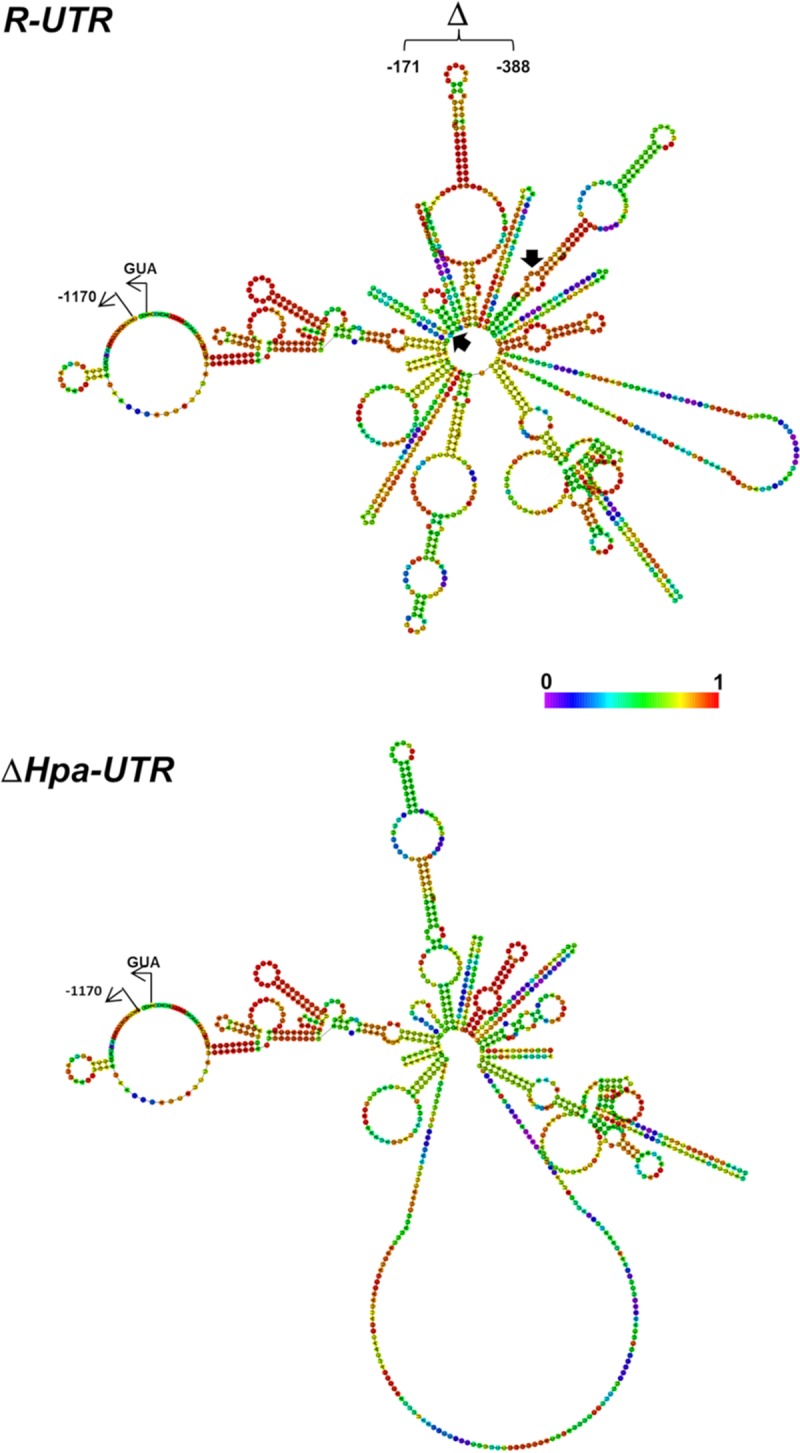
Secondary structure of *EFG1* 5′ UTR. Predicted folding structure of full-length 5′ UTR (*R-UTR*) and deleted 5′ UTR (Δ*Hpa-UTR*) of *EFG1*. The RNAfold program (http://rna.tbi.univie.ac.at/cgi-bin/RNAWebSuite/RNAfold.cgi) was used for prediction, and results were depicted as a centroid structure showing base pair probabilities. The color code indicates probabilities of base-pairing or single-strandedness in predicted paired and unpaired regions, respectively. Black arrows frame the region deleted in the Δ*Hpa-UTR* structure (Fig. S1), which lacks a strong hairpin between positions −327 and −229. Note that strong pairing is predicted between the 5′ end of the UTR (−1118 to −1100) and sequences preceding the AUG translational initiation codon (−112 to −25). This structure is still present in the deleted UTR.

The molecular mechanism by which the 5′ UTR sequences of *EFG1* or *UME6/WOR1* transcripts regulate translation is not known and needs experimental verification. The 218-nt sequence of the *EFG1* 5′ UTR is predicted to form a hairpin (Fig. 8), which possibly could help to generate an mRNA structure that is favorable for translational initiation. This potential structure could also be the target of RNA binding proteins that stimulate translation. For example, unwinding of RNA structures by binding of helicase eIF4A to the 5′ UTR has been reported elsewhere (50). In C. albicans, the Dom34 protein, a predicted component of the no-go transcript degradation pathway, was found to bind to the 5′ UTR of transcripts encoding protein O-mannosyltransferases and to promote their translation (36). Binding proteins could also have an inhibitory function, such as the Rim4 protein in the yeast S. cerevisiae that binds to the 5′ UTR of the *CLB3* transcript to inhibit its translation (51). Likewise, the Ssd1 protein represses translation of genes involved in cell growth and morphogenesis by binding to the 5′ UTR of target transcripts (38). In mammalian cells, glucose-induced translation of insulin requires proteins binding to the 5′ UTR of the encoding transcript (52). On the other hand, the 5′ UTR structures of several human gene transcripts are known to mediate translational control that is essential to prevent several serious diseases (51, 62). The function of 5′ UTR binding proteins is possibly related to the regulation of ribosomal assembly at the AUG initiation codon. Interestingly, the recruitment of regulatory factors to transcripts may depend not only on 5′ UTR or other transcript sequences, since promoters also can provide regulatory proteins that control the degradation, localization, and translation of transcripts (26, 27). It has been suggested that such proteins may be loaded onto the mRNA near its 5′ end early in transcription (28). Such a mechanism could also be operative for the *EFG1* 5′ UTR, because its positive effect was detected only in the context with its native upstream promoter sequences but not with heterologous *PCK1* and *ACT1* promoters, which were able to drive functional expression of the *EFG1* ORF lacking the 5′ UTR (1, 3, 43, 44). Although the functional interplay of promoter and 5′ UTR sequences remains to be established, it is possible that *EFG1* promoter sequences support the action of the 5′ UTR in translation, e.g., by transcript loading with positively acting translation factors. Several other mechanisms explaining the regulatory function of the 5′ UTR sequence in the major *EFG1* transcript are possible (50). Internal ribosome entry sites (IRESs) have been described not only for viral transcripts or genomes but also for translation of yeast genes involved in responses to stress or starvation, which require IRESs within transcripts (34, 53, 54). uORF sequences can occupy 5′ UTRs and contribute to regulation of eukaryotic translation (31, 32). In C. albicans, for example, a uORF regulates translation of the *GCN4* transcript (33). We identified a short uORF with an AUG start codon in the 5′ UTR of *EFG1* in the C. albicans strain ATCC 2013. However, this uORF does not appear to be relevant, since it does not occur in the *EFG1* 5′ UTR of strain SC5314 and its deletion did not influence functional expression of *EFG1* in strain ATCC 2013. However, it should be considered that in the yeast S. cerevisiae translational initiation has been observed also at non-AUG codons, especially at UUG and GUG (55), and the use of GUG for translational initiation in C. albicans has already been reported (56). Interestingly, assuming that UUG can be used for translational initiation in C. albicans, two uORFs placed side by side are predicted within the 218-nt regulatory sequence of the *EFG1* transcript (see Fig. S1 in the supplemental material). These uORFs could potentially encode peptides of 53 and 29 amino acids, respectively. In general, however, uORFs are known to negatively influence the translation of ORF sequences that are situated immediately downstream (31), rather than acting positively as in the case of the *EFG1* ORF. Since all identified uORFs also terminate in the 5′ UTR of *EFG1*, a potential translational read-through generating an extended Efg1 protein, as has been observed for Myc (57), can be excluded. Whatever the underlying mechanism of regulation by 5′ UTR sequences may be, it may be relevant for a significant number of virulence-related C. albicans genes that carry extensive 5′ UTRs. It can also be speculated that such processes may become new targets for antifungal compounds.

## MATERIALS AND METHODS

### Strains and media.

Strains used in this study are listed in Table S1 in the supplemental material. Strains were grown in liquid YP (1% yeast extract, 1% peptone) with either 2% glucose (YPD) or 10% horse serum, to induce filamentation. To induce filamentation on agar, strains were grown on Spider medium (0.3% beef extract, 0.5% peptone, 0.2% K_2_HPO_4_, 1% mannitol, and 2% agar, pH 7.2). An Invivo 200 hypoxia chamber (Ruskinn) was used for hypoxic growth (0.2% O_2_).

10.1128/mSphere.00280-18.2 TABLE S1  Strains. Download TABLE S1, DOCX file, 0.02 MB. Copyright © 2018 Desai et al. 2018 Desai et al. This content is distributed under the terms of the Creative Commons Attribution 4.0 International license.

### Construction of strains containing deletions in the 5′ UTR of *EFG1*.

Expression vector pTD38-HA (46) was modified to remove sequences encoding the N-terminal hemagglutinin (HA) tag, which has been shown to block filamentation phenotypes of Efg1 (8). For this purpose, an AflII restriction site was introduced by site-specific mutagenesis (QuikChange kit; Agilent) using primers MAflIIFor/rev (Table S2) downstream of the HA tag sequence, between positions −7 and −2 bp upstream of the *EFG1* ORF (sequence 5′-ACCCCTTAAGA ATG). The resulting plasmid pPD21HA-AB was cut with PacI and AflII to remove all upstream sequences, which were replaced by a fragment lacking HA sequences generated by PCR using primers 5UTREfgSphIFor/5UTREfgAflIIrev using pTD38-HA as the template. The resulting plasmid pPD21-AB contains 3.2 kb of upstream sequences (comprising 2 kb of promoter and 1.2 kb of 5′ UTR sequences) upstream of the *EFG1* ORF. To delete sequences within the 5′ UTR, novel restriction sites were inserted singly or in combination by site-specific mutagenesis at positions −1167 (SnaBI), −1112 (StuI), −787 (NruI), and −167 (HpaI), using primers listed in Table S2 (Fig. S1). Plasmids were digested pairwise using SnaBI/HpaI, StuI/NruI, NruI/HpaI (native HpaI site at −391), NruI/HpaI (−167), and HpaI (−391)/HpaI (−167) enzymes and religated, to generate plasmids p∆L-UTR, p∆SN-UTR, p∆NH-UTR, p∆NH2-UTR, and p∆Hpa-UTR, respectively. Furthermore, the sequence between HpaI (−167) and position −6 was deleted using primer mutagenesis, to construct plasmid p∆sUTR. Plasmids were linearized with PacI (1.9 kb upstream of *EFG1* ORF) and transformed into strain HLC67 (*efg1* mutant lacking the *EFG1* ORF). The correct integration of the plasmid in the *EFG1* locus was confirmed by colony PCR using primers ColoEfg1For/ColoEfg1Rev.

10.1128/mSphere.00280-18.3 TABLE S2  Oligonucleotides. Download TABLE S2, DOCX file, 0.02 MB. Copyright © 2018 Desai et al. 2018 Desai et al. This content is distributed under the terms of the Creative Commons Attribution 4.0 International license.

### Construction of strains producing click beetle luciferase.

To construct a plasmid carrying a green click beetle luciferase gene with a *sat1* nourseothricin selection marker gene, the plasmid pGEM-HIS1-CBG (47) was restricted with BamHI and MscI (New England BioLabs [NEB]) to cut out and replace the *HIS1* gene sequence. The sequence for the *sat1* marker was obtained from the donor plasmid PFA-SAT1 (58) using the two restriction enzymes PvuII and BamHI. The obtained *sat1* sequence was then integrated into the pGEM plasmid directly downstream of the *CBG* gene via ligation to obtain the plasmid pGEM-SAT1-CBG, which was used as the *CBGluc-sat1* reporter cassette template. Reporter cassettes were amplified via PCR with the primer pairs inACT1-CBG-Fw/inACT1-SAT1-Bw and inEFG1-CBG-Fw/inEFG1-SAT1-Bw (Table S2). These primers carry 60-bp homology to the gene of interest, *ACT1* and *EFG1*, respectively. The DNA fragments were transformed into the parental strains PDUWT (*efg1/R-UTR-EFG1*) and PDUHH (*efg1*/Δ*Hpa-UTR*-*EFG1*). Homologous integration of the luciferase-*sat1*-reporter cassette sequence occurred downstream of the respective start codon of *ACT1* or *EFG1* genes, resulting in 2 reporter strains each for PDUWT (ATC1GN and EFG1GN) and PDUHH (DUTRinACT1GN and DUTRinEFG1GN). Mutants were selected for positive luminescence signals, and correct integration was checked via colony PCR using the primer pairs ACT1 col Fw/CBG col Bw (*ACT1*) and EFG1 col Fw/CBG col Bw (*EFG1*). Mutants positive for both colony PCR and luminescence were used for further experiments.

### Blotting procedures.

For Northern blotting assays, the strains were grown at 30°C to the logarithmic phase, total RNA was isolated, and 8 µg of RNA was separated on agarose gels containing 1.2% formaldehyde. Following transfer to nylon membranes (Roche), blots were hybridized with ^32^P-labeled probes for *EFG1* using primers ProFor and ProRev. For signal detection, the washed membranes were exposed to phosphor screen (Fujifilm) for 30 to 60 min and scanned by the FLA 5000 phosphorimager (Fujifilm).

For immunoblotting assays, YPD precultures grown overnight at 30°C in YPD medium were used to inoculate 30 ml of YPD medium. Strains were grown to an optical density at 600 nm (OD_600_) of 0.1, harvested by centrifugation, frozen at −70°C for 1 h, and then thawed by addition of 500 ml of CAPSO buffer (20 mM *N*-cyclohexyl-2-hydroxyl-3-aminopropanesulfonic acid [CAPSO], pH 9.5, 1 M NaCl, 1 mM EDTA, 20 mM imidazole, 0.1% Triton X-100) containing protease inhibitor (cOmplete cocktail, Mini, EDTA-free; Roche). Cell extracts were prepared as described previously (8). Eighty micrograms of the crude cell extract was separated by SDS-PAGE (10% polyacrylamide) and analyzed by immunoblotting using anti-Efg1 antiserum (1:5,000) (8) or anti-histone H4 (Abcam; 1:5,000) to detect histone H4 as a loading control. Anti-rabbit-IgG-horseradish peroxidase (HRP) conjugate (1:10,000) was used as secondary antibody in all blotting assays. Signals generated by the chemiluminescent substrate (SuperSignal West Dura; Pierce) were detected by a LAS-4000 mini-imager (Fujifilm) and evaluated by Multi Gauge software (Fujifilm).

### Polysome profiling.

C. albicans strains PDUWT and PDUHH were grown exponentially in YPD medium to an OD_600_ of 0.4 to 0.6. For preparation of samples derived from cells following hyphal induction, exponentially growing cells were washed with 1× phosphate-buffered saline (PBS), resuspended in YP medium containing 10% horse serum (prewarmed at 37°C), and incubated at 37°C for 15 min. Preparation of cells for polysome gradients was performed as described previously (36, 59), with some modifications. A portion of the culture (80 ml) was recovered and chilled for 5 min on ice in the presence of 0.1 mg/ml cycloheximide (CHX). Cells were harvested by centrifugation at 6,000 × *g* for 4 min at 4°C and resuspended in lysis buffer (20 mM Tris-HCl, pH 8, 140 mM KCl, 5 mM MgCl_2_, 0.5 mM dithiothreitol, 1% Triton X-100, 0.1 mg/ml cycloheximide, and 0.5 mg/ml heparin). After washing, cells were resuspended in 700 µl of lysis buffer, 300 µl glass beads was added, and cells were disrupted by shaking on a Vortex Genie 2 (setting 8) using 6 cycles for 40 s. Between cycles, cells were placed on ice for 5 min. Lysates were cleared by centrifuging twice for 5 min, first at 5,000 rpm and then at 8,000 rpm for the recovered supernatant. Finally, glycerol was added to the supernatant at a final concentration of 5% before storing extracts at −70°C. Samples of 10 to 20 *A*_260_ units were loaded onto 10 to 50% sucrose gradients and were separated by ultracentrifugation for 2 h 40 min at 35,000 rpm in a Beckman SW41 rotor at 4°C. Then, gradients were fractionated using isotonic pumping of 60% sucrose from the bottom, followed by recording of the polysomal profiles by online UV detection at 254 nm (density gradient fractionation system; Teledyne ISCO, Lincoln, NE). To analyze the RNA of the polysomal fractions, RNA from 200 µl of each fraction was extracted using the GeneJET RNA extraction kit (Strek; Biotools). To each sample, 500 ng of *in vitro*-transcribed RNA (HiScribe T7 high-yield RNA synthesis kit; NEB) was added and used as spiked-in mRNA for normalization of the transcripts. After reverse transcription of the purified RNA (Maxima first-strand cDNA synthesis kit; Thermo Scientific), quantitative PCR (qRT-PCR) was performed using gene-specific primer pairs to quantify mRNAs of *EFG1* and *ACT1*. For each fraction, two biological replicates with three technical replicates were assayed on an Mx3000P Light Cycler (Stratagene), with 10 µl of cDNA, 4 µl EvaGreen qPCR mix II (Bio-Budget), and 3 µl each of forward and reverse oligonucleotide primers (400 pmol/µl) in each reaction mixture. The polymerase was activated at 95°C for 10 min, annealing was performed at 60°C for 15 s, extension was performed at 72°C for 30 s, and the denaturation step was performed at 95°C for 30 s, using a total of 50 cycles.

### qRT-PCR.

cDNA for qRT-PCR analysis was prepared from 2 µg of total RNA treated with DNase I (Thermo Fisher) using the Maxima first-strand cDNA synthesis kit (Thermo Fisher). Real-time PCR was performed in triplicate in 96-well plates using the EvaGreen dye (Bio-Budget). Primers used for qRT-PCR analysis are described in Table S2. Real-time PCR was performed using the following cycling conditions: step 1, 95°C for 15 min; step 2, 95°C for 15 s; step 3, annealing temperature of 60°C for 20 s; step 4, elongation, 72°C for 20 s; step 5, repeat of steps 2 to 4 39 times; step 7, melting curve of 50°C to 95°C every 0.4°C, hold for 1 s, and reading of plate. Expression levels of each gene were normalized to levels of an internal *ACT1* control using the Pfaffl method (60).

### Luciferase assay.

To measure click beetle luciferase activity in yeast cells, overnight cultures of PDUWT, PDUHH, ACT1GN, EFG1N, UTRinACT1GN, and UTRinEFG1GN were diluted to an OD_600_ of 1.0 in PBS buffer (140 mM NaCl, 3 mM KCl, 8 mM Na_2_HPO_4_, 1.8 mM KH_2_PO_4_, pH 7.4) and incubated at 30°C for 60 min at 180 rpm. One milliliter was transferred into fresh YPD medium and grown for 6 h at 30°C. All samples were set to an OD_600_ of 0.3 in 1 ml YPD and quickly frozen in liquid nitrogen. After thawing, 100 µl of the samples was transferred into a 96-well microtiter plate, and 100 µl Beetleglow (47) was added to start the reaction. Measurements were made in an Infinite M200 Pro plate reader (Tecan) with the following settings. Plates were shaken for 10 s at 140 rpm, and relative luminescence units (RLU) were measured for 1 s per well at 30°C. Each plate was measured 3 times, and the maximal luminescence values (*L*_max_) were reported.

### Data availability.

All data are included in the article and supplemental files.
