# Supplementary figures and images for: The 5′ Untranslated Region of the EFG1 Transcript Promotes Its Translation To Regulate Hyphal Morphogenesis in Candida albicans
Source: mSphere. 2018 Jul 5;3(4):e00280-18. doi: 10.1128/mSphere.00280-18 (PMC6034079; doi:10.1128/mSphere.00280-18)

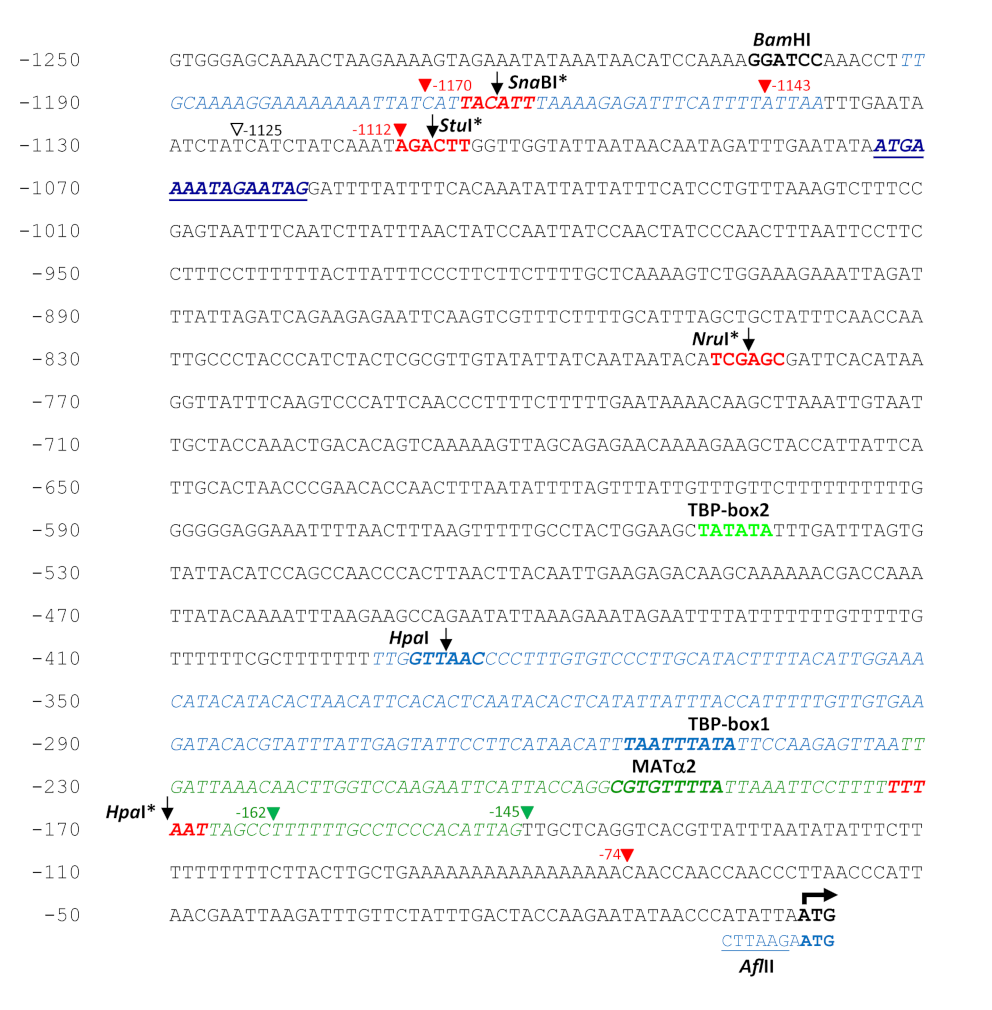

Supplement: FIG S1 [file sph003182578sf1.tif]
