# Supplementary material for: The 5′ Untranslated Region of the EFG1 Transcript Promotes Its Translation To Regulate Hyphal Morphogenesis in Candida albicans
Source: mSphere. 2018 Jul 5;3(4):e00280-18. doi: 10.1128/mSphere.00280-18 (PMC6034079; doi:10.1128/mSphere.00280-18)
Supplement: TABLE S1 [file sph003182578st1.docx]

**Table S1. Strains**

| **Strain Name** | **Genotype** | **Reference** |
| --- | --- | --- |
| CAF2-1 | *ura3*Δ::*imm*434/*URA3* | [1] |
| CAI4 | *ura3*Δ::*imm434*/*ura3*Δ::*imm434* | [1] |
| BCA0901 | Like *CAI4* but *efg1/efg1::hisG-URA3-hisG* | [2] |
| HLC67 | Like *CAI4* but *efg1::hisG/efg1::hisG* | [3] |
| HLCEEFG1 | Like HLC67, but *efg1*::*hisG*/*efg1*::[*EFG1p-HA-EFG1-URA3*]  (pTD38-HA/*Pac*I integrated in *EFG1* promoter) | [4] |
| PDUWT | Like HLC67, but *efg1*::*hisG*/*efg1*::[*EFG1p-R-UTR-EFG1 URA3*]  (pPD21-AB/*Pac*I integrated in *EFG1* promoter)  Full length 5’ UTR with no deletions and without HA-tag (*R-UTR*) | This study |
| PDULG | Like HLC67, but *efg1*::*hisG*/*efg1*::[*EFG1p*-Δ*L-UTR-EFG1 URA3*]  (p∆L-UTR/*Pac*I integrated in *EFG1* promoter) Deletion in 5’ UTR from -1164 bp to -171 bp (Δ*L-UTR*) | This study |
| PDUSN | Like HLC67, but *efg1*::*hisG*/*efg1*::[*EFG1p*-ΔSN*-UTR-EFG1 URA3*]  (p∆SN-UTR/*Pac*I integrated in *EFG1* promoter).  Deletion in 5’ UTR from -1109 bp to -785 bp (Δ*SN-UTR*) | This study |
| PDUNH | Like HLC67, but *efg1*::*hisG*/*efg1*::[*EFG1p*-Δ*NH-UTR-EFG1 URA3*] (p∆NH-UTR/*Pac*I integrated in *EFG1* promoter)  Deletion in 5’ UTR from -784 bp to -389 bp (Δ*NH-UTR*) | This study |
| PDUSH | Like HLC67, but *efg1*::*hisG*/*efg1*::[*EFG1p*-ΔNH2*-UTR-EFG1-URA3*]  (p∆NH2-UTR/*Pac*I integrated in *EFG1* promoter)  Deletion in 5’ UTR from -784 bp to -171 bp (Δ*NH2-UTR*) | This study |
| PDUHH | Like HLC67, but *efg1*::*hisG*/*efg1*::[*EFG1p*-ΔHpa*-UTR-EFG1 URA3*]  (p∆Hpa-UTR/*Pac*I integrated in *EFG1* promoter)  Deletion in 5’ UTR from -388 bp to -171 bp (Δ*Hpa-UTR*) | This study |
| PDUsU | Like HLC67, but *efg1*::*hisG*/*efg1*::[*EFG1p*-ΔsUTR*-EFG1 URA3*]  (p∆sUTR/*Pac*I integrated in *EFG1* promoter)  Deletion in 5’ UTR from -167 bp to -1 bp (Δ*sUTR*) | This study |
| ACT1GN | Like PDUWT, but *ACT1/act1*::[*ACT1p*-*CaCBGluc sat1*] | This study |
| EFG1GN | Like PDUWT, but *efg1/efg1*::[*EFG1p*-*R-UTR-CaCBGluc sat1*] | This study |
| DUTRinACT1GN | Like PDUHH, but *ACT1/act1*::[*ACT1p*-Ca*CBGluc sat1*] | This study |
| DUTRinEFG1GN | Like PDUHH, but *efg1/efg1*::[*EFG1p*-Δ*Hpa-UTR-CaCBGluc sat1*] | This study |

**References for Table S1.**

1. Fonzi WA, Irwin MY. 1993. Isogenic strain construction and gene mapping in *Candida albicans*. Genetics 134:717-728
2. Braun BR, Johnson AD. 2000. *TUP1*, *CPH1* and *EFG1* make independent contributions to filamentation in *Candida albicans*. Genetics 155: 57-67
3. Lo HJ, Köhler JR, DiDomenico B, Loebenberg D, Cacciapuoti A, Fink GR. 1997. Nonfilamentous *C. albicans* mutants are avirulent. Cell 90:939-949
4. Noffz CS, Liedschulte V, Lengeler K, Ernst JF. 2008. Functional mapping of the *Candida albicans* Efg1 regulator. Eukaryot Cell 7:881-893
