# Supplementary material for: The 5′ Untranslated Region of the EFG1 Transcript Promotes Its Translation To Regulate Hyphal Morphogenesis in Candida albicans
Source: mSphere. 2018 Jul 5;3(4):e00280-18. doi: 10.1128/mSphere.00280-18 (PMC6034079; doi:10.1128/mSphere.00280-18)
Supplement: TABLE S2 [file sph003182578st2.docx]

**Table S2. Oligonucleotides**

| Primer Name | Sequence^1^ |
| --- | --- |
| DUTR/TS | 5´-GGA AAA AAA ATT ATC AT**T ACG TA**T AAA AGA G (*Sna*BI) |
| iDUTR/TS | 5´-CTC TTT TA**T ACG TA**A TGA TAA TTT TTT TTC C (*Sna*BI) |
| UTR-STU1 | 5´-CTA TCA AAT **AGG CCT** GGT TGG (*Stu*I) |
| UTR-STU2 | 5´-CCA ACC **AGG CCT** ATT TGA TAG (*Stu*I) |
| UTR-NRU1 | 5´-CAA TAA TAC A**TC GCG A**GA TTC AC (*Nru*I) |
| UTR-NRU2 | 5´-GTG AAT C**TC GCG A**TG TAT TAT TG (*Nru*I) |
| UTR-HPA1 | 5´-CCT TTT **GTT AAC** TAG CCT TTT TTG C (*Hpa*I) |
| UTR-HPA2 | 5´-GCA AAA AAG GCT A**GT TAA C**AA AAG G (*Hpa*I) |
| MSnaB1For | 5’-GGAAAAAAAATTATCAT**TACGTA**TAAAAGAG-3’ (*Sna*BI) |
| MSnaB1Rev | 5’-CTCTTTTA**TACGTA**ATGATAATTTTTTTTC C-3’ (*Sna*BI) |
| MStuIfor | 5’-CTATCAAAT**AGGCCT**GGTTGG-3’ (*Stu*I) |
| MStuIRev | 5’-CCAACC**AGGCCT**ATTTGATAG-3’ (*Stu*I) |
| MNruIFor | 5’-CAATAATACA**TCGCGA**GATTCAC-3’ (*Nru*I) |
| MNruIRev | 5’-GTGAATC**TCGCGA**TGTATTAT TG-3’ (*Nru*I) |
| MHpaIFor | 5’-CCT TTT**GTTAAC**TAGCCTTTTTTGC-3’ (*Hpa*I) |
| MHpaIRev | 5’-GCAAAAAAGGCTA**GTTAAC**AAAAGG-3’ (*Hpa*I) |
| MAflIIFor | 5’-GAATTCCCCGGGCTGCAG**CTTAAG**TA*ATG*TCAACGTATTC-3’ (*Afl*II) |
| MAflIIRev | 5’-GAATACGTTGACATTA**CTTAAG**CTGCAGCCCGGGGAATTC-3’ (*Afl*II) |
| ColoEfg1For | 5’-GAACAGATAGACTCTGTGCATTG -3’ |
| ColoEFG1Rev | 5’-GACCGGACTAGTGGTGGAACCTGCACC-3’ |
| 5UTREfgSphIFor | 5’-CATTCG**GCATGC**GCAGTTGTTATTGAC -3’ |
| 5UTREfgAflIIrev | 5’-CATTGC**CTTAAG**GGGTTATATTCTTGG -3’ |
| AflIIrev | 5’-CATTGC**CTTAAG**CTGGGTTAACAAAAGGAAT-3’ |
| EFG1RTFor | 5’-TAACGGAACCAAATTGCTCA -3’ |
| EFG1RTRev | 5’-CTTTCAAATGCATTGATCCG -3’ |
| ACT1RTFor | 5’-TTGGATTCTGGTGATGGTGT -3’ |
| ACT1RTRev | 5’-TGGACAAATGGTTGGTCAAG-3’ |
| inACT1-CBG-Fw | GACGACGCTCCAAGAGCTGTTTTCCCATCTCTTGTTGGTAGACCAAGACATCAAGGTATCATGGTTAAGAGAGAAAAAAACG |
| inACT1-SAT1-Bw | TCTTCTGGAGCAACTCTCAATTCATTGTAAAAAGTGTGATGCCAGATTTTTTCCATATCGGTCGAGCGTCAAAACTAGAG |
| inEFG1-CBG-Fw | ATGTCAACGTATTCTATACCCTATTACAATCAAATGAACGGAAATTACAATAACGGTATGATGGTTAAGAGAGAAAAAAACG |
| inEFG1-SAT1-Bw | TGTTGACCTGGTTGTCCTTGTTGTTGATAGAACATGTAGTTATAAGGCTGTTGGACTGGTCGAGCGTCAAAACTAGAGAA |
| CBG col Bw | TGGAGCAACTTGTGAACC |
| ACT1 col Fw | CAATATCCTATGGCCAAGGG |
| EFG1 col Fw | TTGCCCTACCCATCTACTCG |
| CBG rt Fw | TTGCATTTCGATCCAGTTGA |
| CBG rt Bw | GAAACCGAAAGCATGGAAGA |
| EF1B-F | AGTCATTGAACGAATTCTTGGCTG |
| EF1B-R | TCTTCATCAACTTCATCATCAGAACC |
| TDH1-F | ATCCCACAAGGACTGGAGA |
| TDH3-R | GCAGAAGCTTTAGCAACGTG |
| ACT1-F | TCAGACCAGCTGATTTAGGTTTG |
| ACT1-R | GTGAACAATGGATGGACCAG |

^1^Introduced restriction sites (in brackets) are marked in bold font; underlines indicate sequences used for homologous chromosomal integration
